# Supplementary material for: TEQUILA-seq: a versatile and low-cost method for targeted long-read RNA sequencing
Source: Nat Commun. 2023 Aug 8;14:4760. doi: 10.1038/s41467-023-40083-6 (PMC10409798; doi:10.1038/s41467-023-40083-6)
Supplement: Supplementary file 1 — Supplementary Information [file 41467_2023_40083_MOESM1_ESM.pdf]

Supplementary Information

TEQUILA-seq: A versatile and low-cost method for targeted long-read RNA sequencing

Feng Wang[1,6], Yang Xu[1,2,6], Robert Wang[1,2,6], Beatrice Zhang[1], Noah Smith[1], Amber Notaro[1], Samantha Gaerlan[1], Eric Kutschera[1], Kathryn E. Kadash-Edmondson[1], Yi Xing[1,3,4,*], Lan Lin[3,5,*]

[1]Center for Computational and Genomic Medicine, Children's Hospital of Philadelphia, Philadelphia, PA, USA

[2]Graduate Group in Genomics and Computational Biology, University of Pennsylvania, Philadelphia, PA, USA

[3]Department of Pathology and Laboratory Medicine, University of Pennsylvania Perelman School of Medicine, Philadelphia, PA, USA

[4]Department of Biomedical and Health Informatics, Children's Hospital of Philadelphia, Philadelphia, PA, USA

[5]Raymond G. Perelman Center for Cellular and Molecular Therapeutics, Children's Hospital of Philadelphia, Philadelphia, PA, USA

[6]These authors contributed equally: Feng Wang, Yang Xu, Robert Wang.

*Corresponding authors. E-mail: xingyi@chop.edu; linlan@chop.edu.

Supplementary Figure 1.

Pairwise comparisons of estimated abundances for transcript isoforms of target genes across TEQUILA-seq and xGen Lockdown-seq libraries.

Supplementary Figure 2.

Estimated abundances of 10 target genes across TEQUILA-seq, xGen Lockdown-seq, and whole-transcriptome nanopore 1D cDNA sequencing (non-capture control) libraries.

Supplementary Figure 3.

Target enrichment of 468 actionable cancer genes in 4 breast cancer cell lines using TEQUILA-seq.

Supplementary Figure 4.

An *FGFR2* transcript isoform with a mutually exclusive exon is the predominant splice isoform in basal B breast cancer cell lines.

Supplementary Figure 5.

An *SESN1* transcript isoform with a distal alternative first exon is the predominant splice isoform in basal B breast cancer cell lines.

Supplementary Figure 6.

Identification of tumor aberrant transcript isoforms across 40 breast cancer cell lines.

Supplementary Figure 7.

A novel aberrant *NOTCH1* transcript isoform resulting from a genomic deletion containing exons 2-27 is the predominant isoform in the MDA-MB-157 cell line.

Supplementary Figure 8.

A novel aberrant *RB1* transcript isoform resulting from a genomic deletion containing exon 22 is the predominant isoform in the HCC1937 cell line.

Supplementary Figure 9.

Confirmation of a splice site-disrupting mutation causing *TP53* splice variants in the HCC1599 cell line.

Supplementary Figure 10.

Elevated transcript expression levels of two PTC-containing transcript isoforms of *TP53* upon NMD inhibition in HCC1599 cells, as indicated by RT-PCR analysis.

Supplementary Figure 11.

Probe yield of individual synthesis reactions measured by Nanodrop.

Supplementary Figure 12.

Target enrichment of 468 actionable cancer genes in 4 breast cancer cell lines using TEQUILA-seq with or without blocking oligos.

Supplementary Figure 13.

Barcoding and multiplexing TEQUILA-seq libraries using the nanopore native barcoding kit.

Supplementary Figure 1

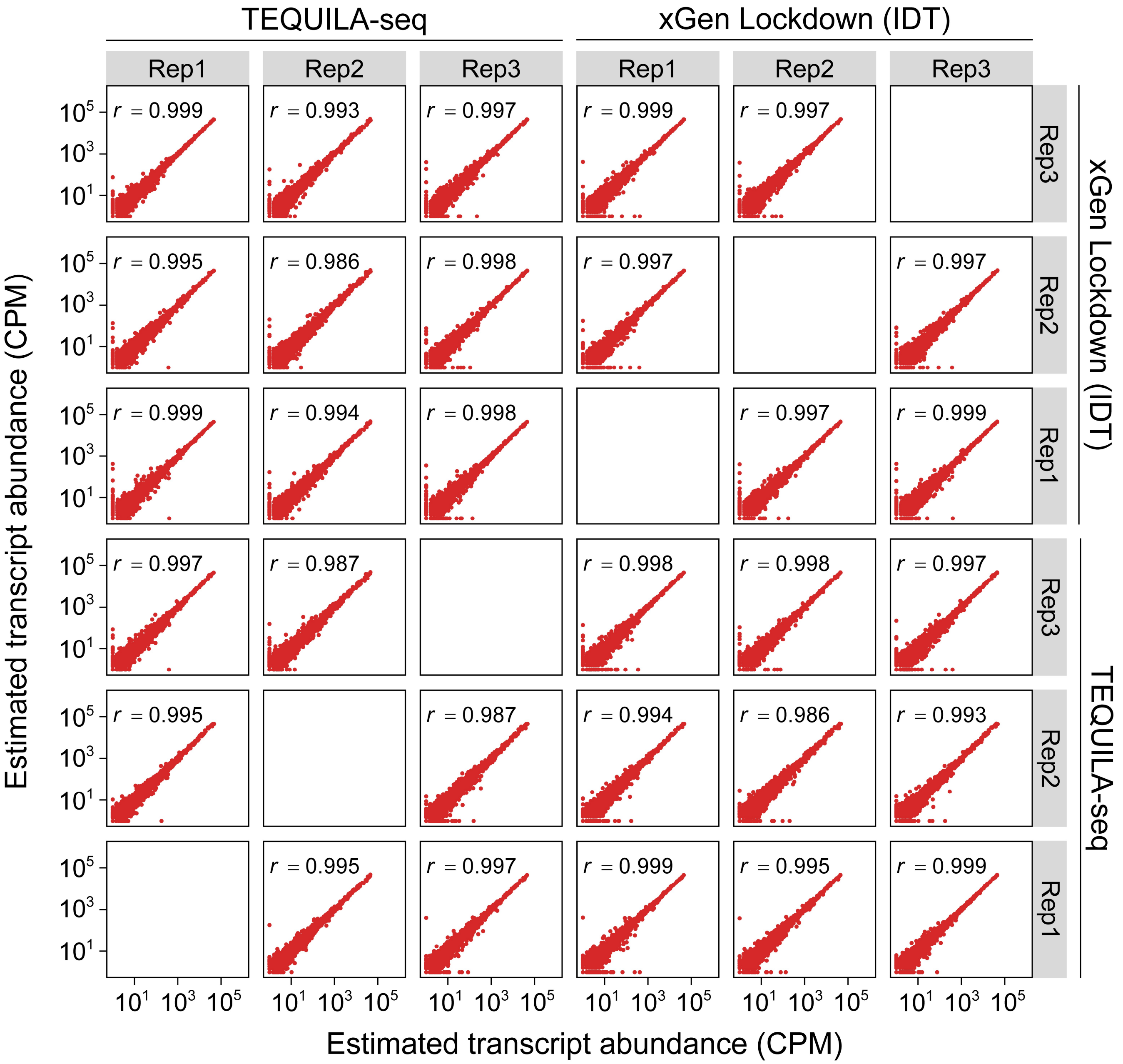

Supplementary Figure 1. Pairwise comparisons of estimated abundances for transcript isoforms of target genes across TEQUILA-seq and xGen Lockdown-seq libraries. For each pairwise comparison, transcript isoforms of target genes with a CPM > 0 in at least one library were plotted and used to calculate the Pearson's correlation. Target gene panel: 10 human genes with long transcripts in the brain. All sequencing methods were applied to the same human brain RNA mix from multiple donors (see Methods).

Supplementary Figure 2

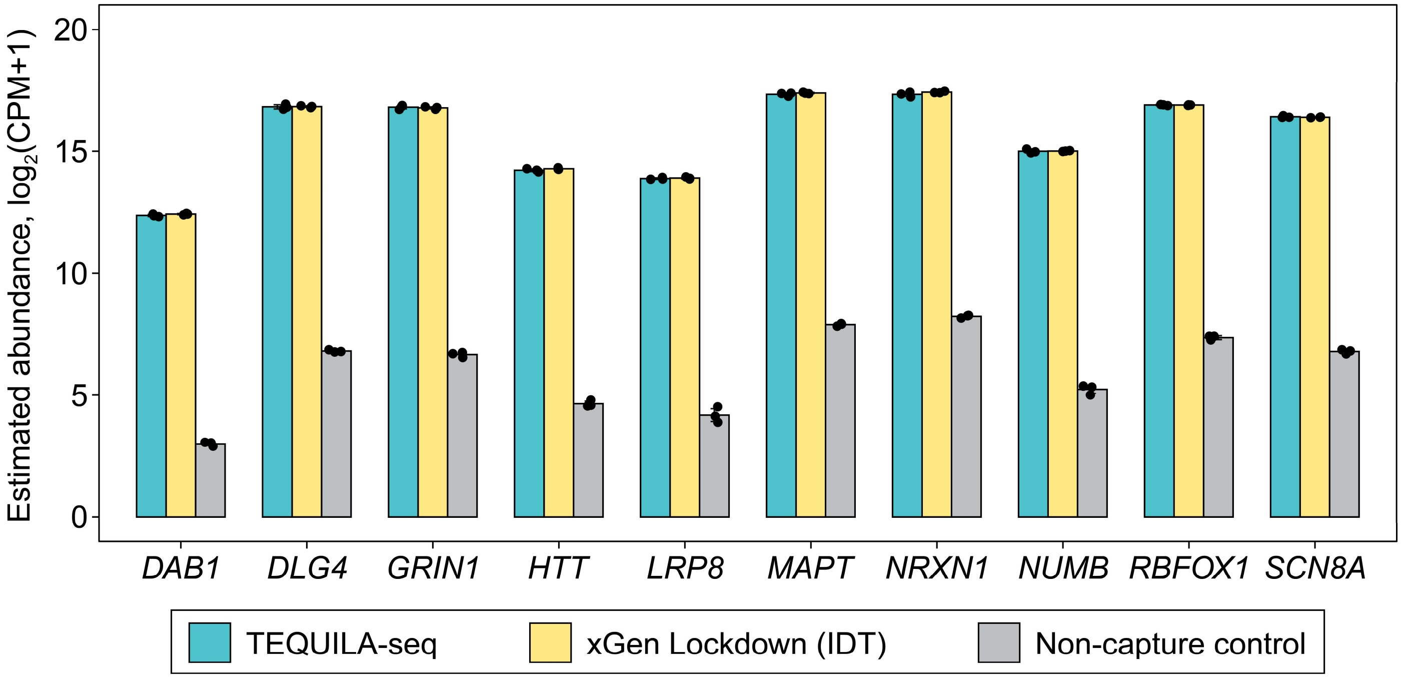

Supplementary Figure 2. Estimated abundances of 10 target genes across TEQUILA-seq, xGen Lockdown-seq, and whole-transcriptome nanopore 1D cDNA sequencing (non-capture control) libraries. Estimated abundance is shown as the mean ± s.d of $n = 3$ replicates.

Supplementary Figure 3

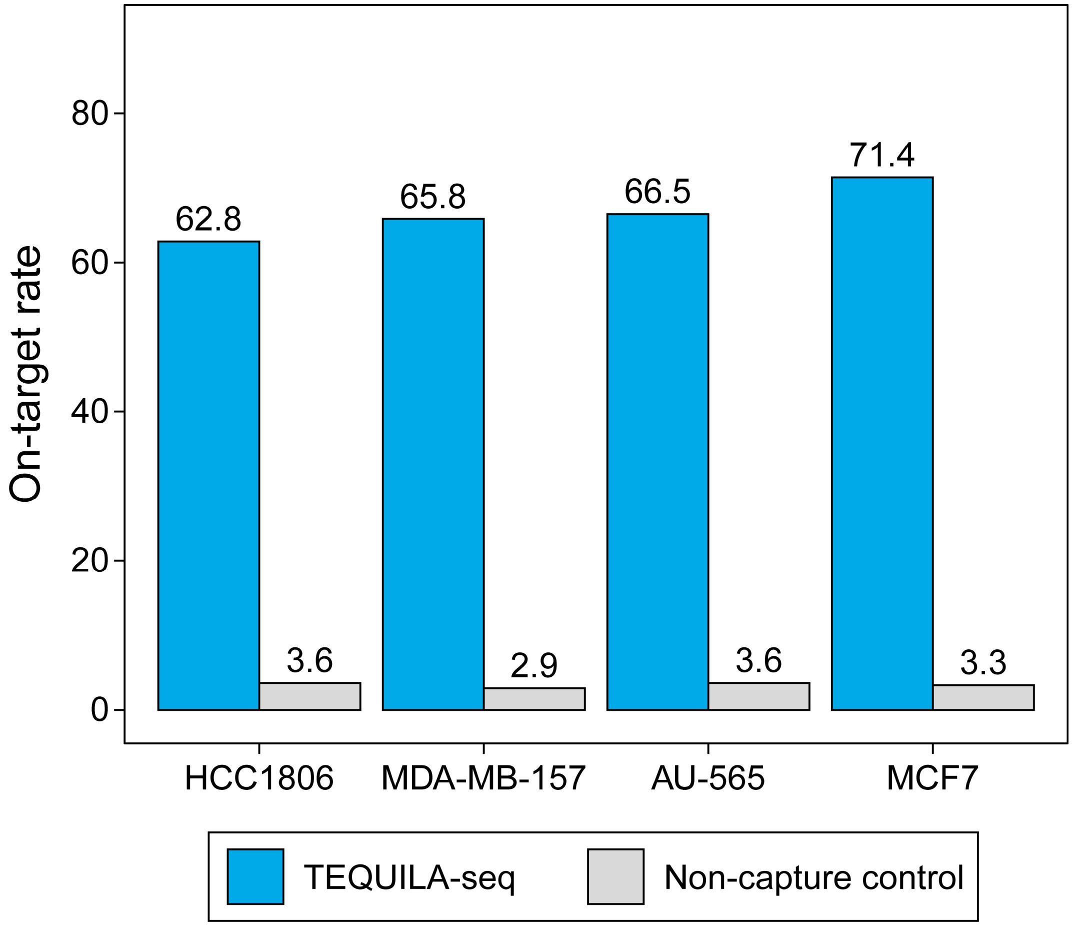

Supplementary Figure 3. Target enrichment of 468 actionable cancer genes in 4 breast cancer cell lines using TEQUILA-seq. For each cell line, TEQUILA-seq and whole-transcriptome nanopore 1D cDNA sequencing (non-capture control) libraries were prepared from the same biological replicate. Each bar shows the percentage of reads mapped to the 468 genes.

Supplementary Figure 4

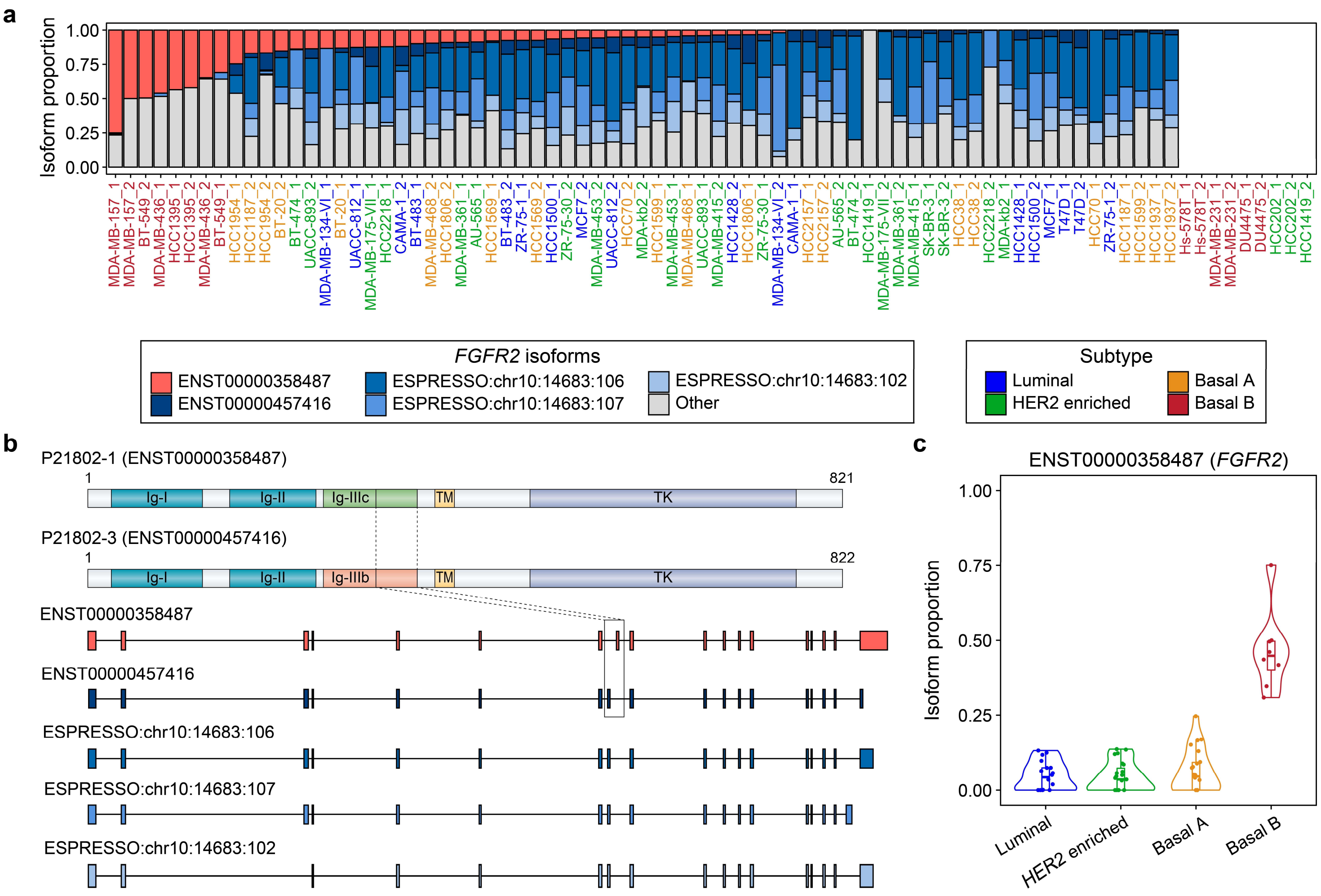

Supplementary Figure 4. An *FGFR2* transcript isoform with a mutually exclusive exon is the predominant splice isoform in basal B breast cancer cell lines. a, Stacked barplots showing proportions of *FGFR2* transcript isoforms discovered by TEQUILA-seq in 40 cell lines. Red bar: isoform of interest (ENST00000358487); navy bar: canonical isoform (ENST00000457416); lighter blue bars: 3 other most abundant *FGFR2* isoforms; gray bars: remaining *FGFR2* isoforms. **b,** Structures of *FGFR2* protein and transcript isoforms. (Upper) Domain annotations for protein isoforms. Immunoglobulin loop domains (Ig-I, Ig-II, and Ig-III); transmembrane domain (TM); tyrosine kinase domain (TK). (Lower) Transcript structures. Boxes: exons. Line segments: introns. **c,** Violin plots (median, interquartile range) showing distribution of isoform proportions for the *FGFR2* isoform of interest (ENST00000358487) in different breast cancer intrinsic subtypes. Each data point represents the isoform proportion in a given cell line replicate (n = 18 for Luminal, n = 26 for HER2 enriched, n = 22 for Basal A, and n = 14 for Basal B).

Supplementary Figure 5

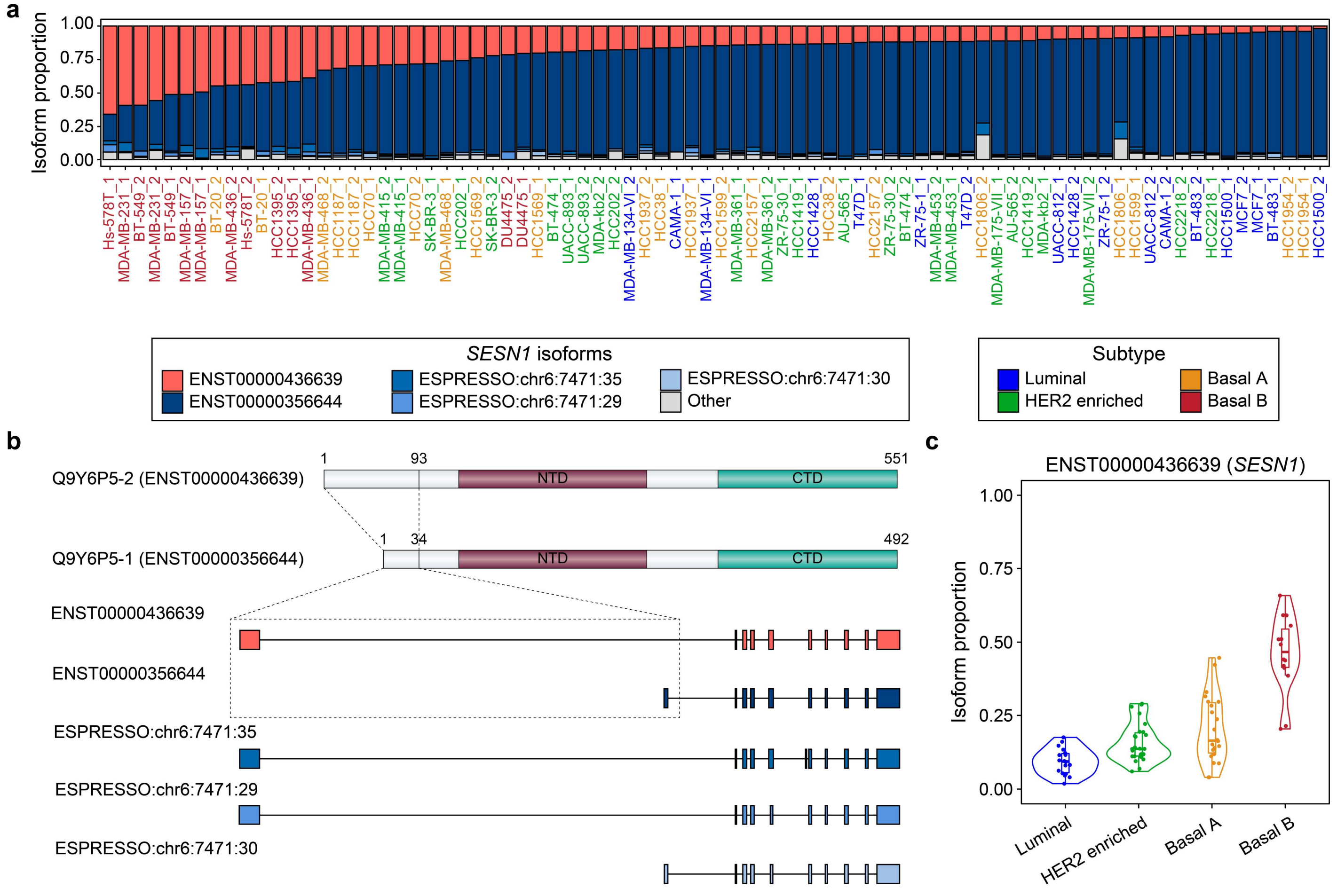

Supplementary Figure 5. An *SESN1* transcript isoform with a distal alternative first exon is the predominant splice isoform in basal B breast cancer cell lines. a, Stacked barplots showing proportions of *SESN1* transcript isoforms discovered by TEQUILA-seq in 40 cell lines. Red bar: isoform of interest (ENST00000436639); navy bar: most abundant annotated protein-coding isoform (ENST00000356644); lighter blue bars: 3 other most abundant *SESN1* isoforms; gray bars: remaining *SESN1* isoforms. **b,** Structures of *SESN1* protein and transcript isoforms. (Upper) Domain annotations for protein isoforms. N-terminal domain (NTD); C-terminal domain (CTD). (Lower) Transcript structures. Boxes: exons. Line segments: introns. **c,** Violin plots (median, interquartile range) showing distribution of isoform proportions for the *SESN1* isoform of interest (ENST00000436639) in different breast cancer intrinsic subtypes. Each data point represents the isoform proportion in a given cell line replicate (n = 18 for Luminal, n = 26 for HER2 enriched, n = 22 for Basal A, and n = 14 for Basal B).

Supplementary Figure 6

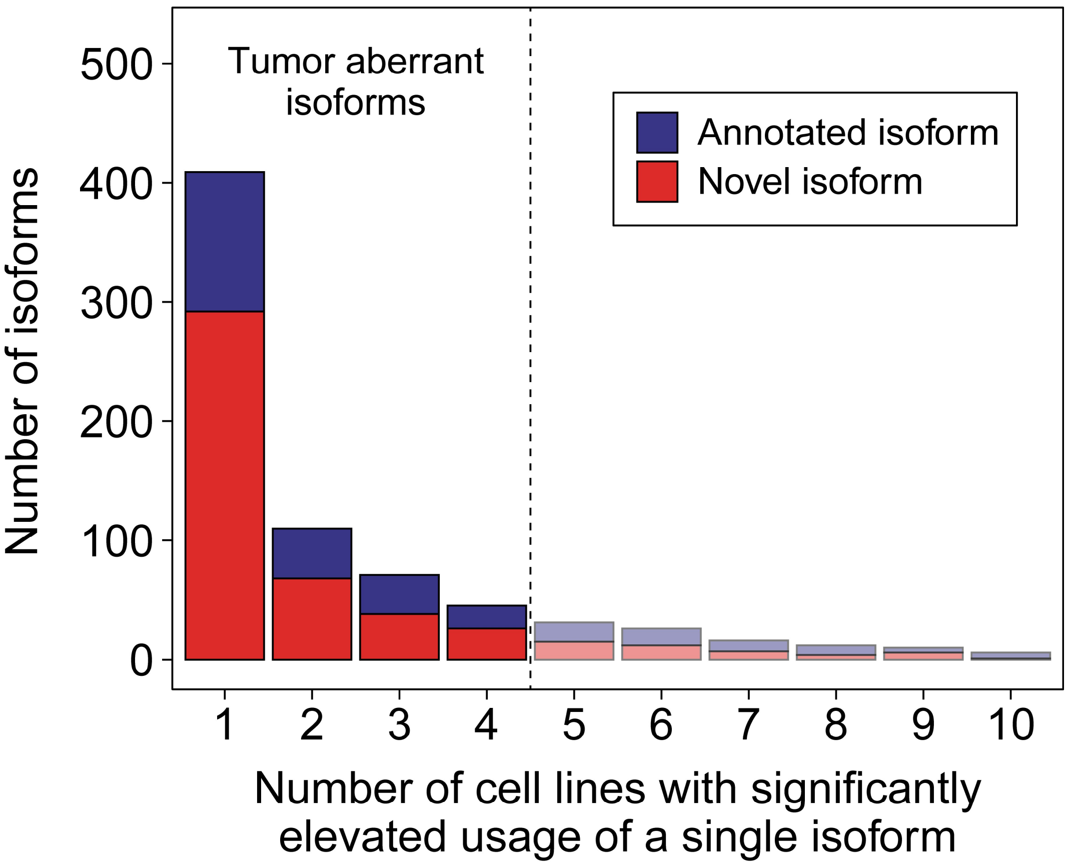

Supplementary Figure 6. Identification of tumor aberrant transcript isoforms across 40 breast cancer cell lines. Stacked barplots showing the number of transcript isoforms with significantly elevated usage in at least one cell line (see Methods) as a function of the corresponding number of such cell lines. Dashed line: cutoff for defining “tumor aberrant” transcript isoforms.

Supplementary Figure 7

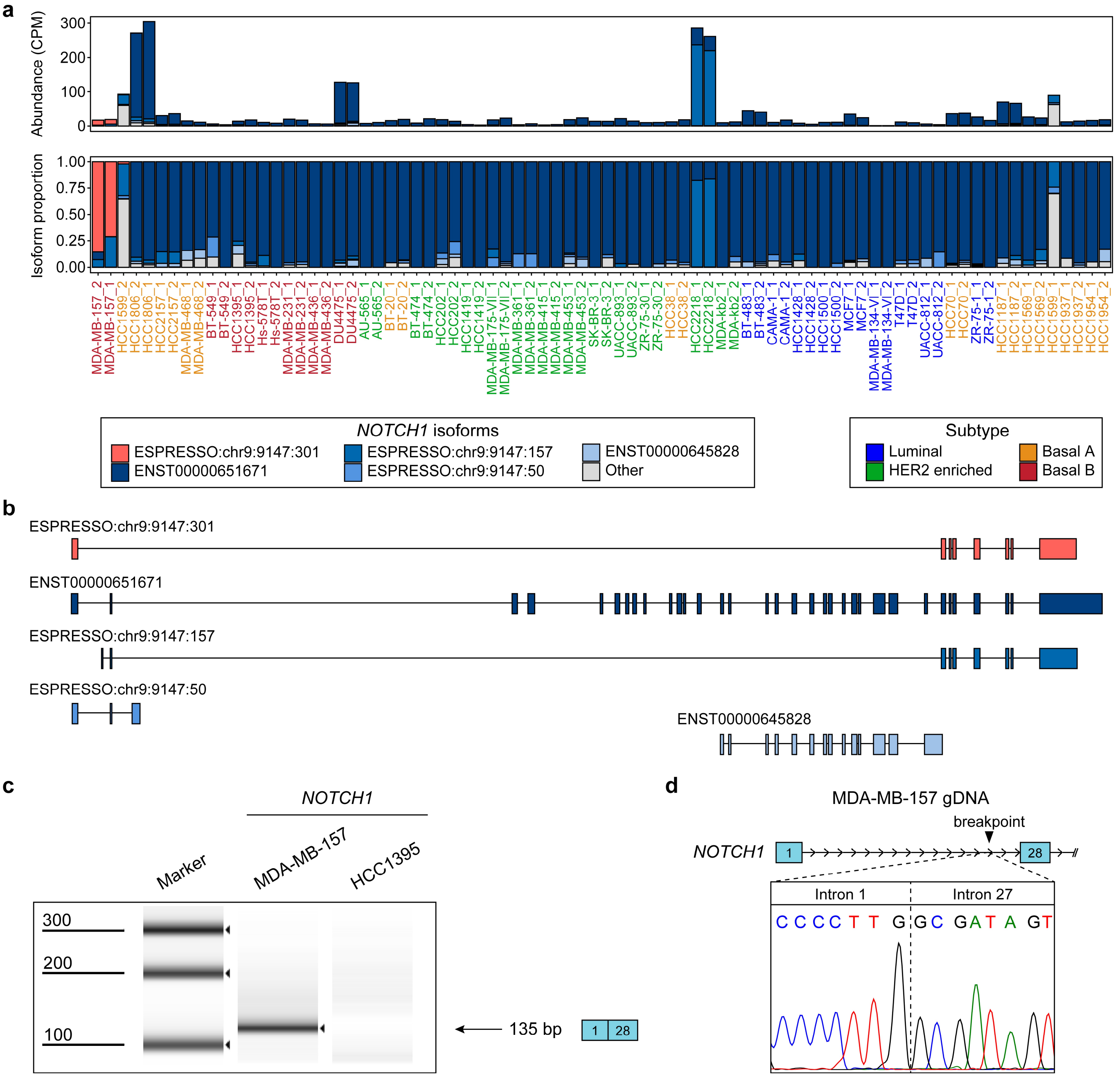

Supplementary Figure 7. A novel aberrant *NOTCH1* transcript isoform resulting from a genomic deletion containing exons 2-27 is the predominant isoform in the MDA-MB-157 cell line. a, Stacked barplots showing estimated abundances (upper panel) and proportions (lower panel) of *NOTCH1* transcript isoforms discovered by TEQUILA-seq in 40 cell lines. Red bar: isoform of interest (ESPRESSO:chr9:9147:301), navy bar: canonical isoform (ENST00000651671); lighter blue bars: 3 other most abundant *NOTCH1* isoforms; gray bars: remaining *NOTCH1* isoforms. **b,** Structures of *NOTCH1* transcript isoforms. Boxes: exons. Line segments: introns. **c,** RT-PCR validation of the splice junction between exons 1 and 28 of *NOTCH1* in MDA-MB-157 and HCC1395 (control) cell lines. The 135-bp product corresponds to the predominant novel transcript isoform resulting from skipping of exons 2 to 27 in MDA-MB-157. Each RT-PCR was repeated twice and yielded the same PCR products. **d,** Sanger sequencing identifies a ~41.5 kb genomic deletion in MDA-MB-157. Sequencing results for the *NOTCH1* gDNA amplicons from MDA-MB-157 are shown. Breakpoints of the deletion are located in introns 1 and 27 of *NOTCH1*. Dashed line in partial sequence chromatogram: breakpoint.

Supplementary Figure 8

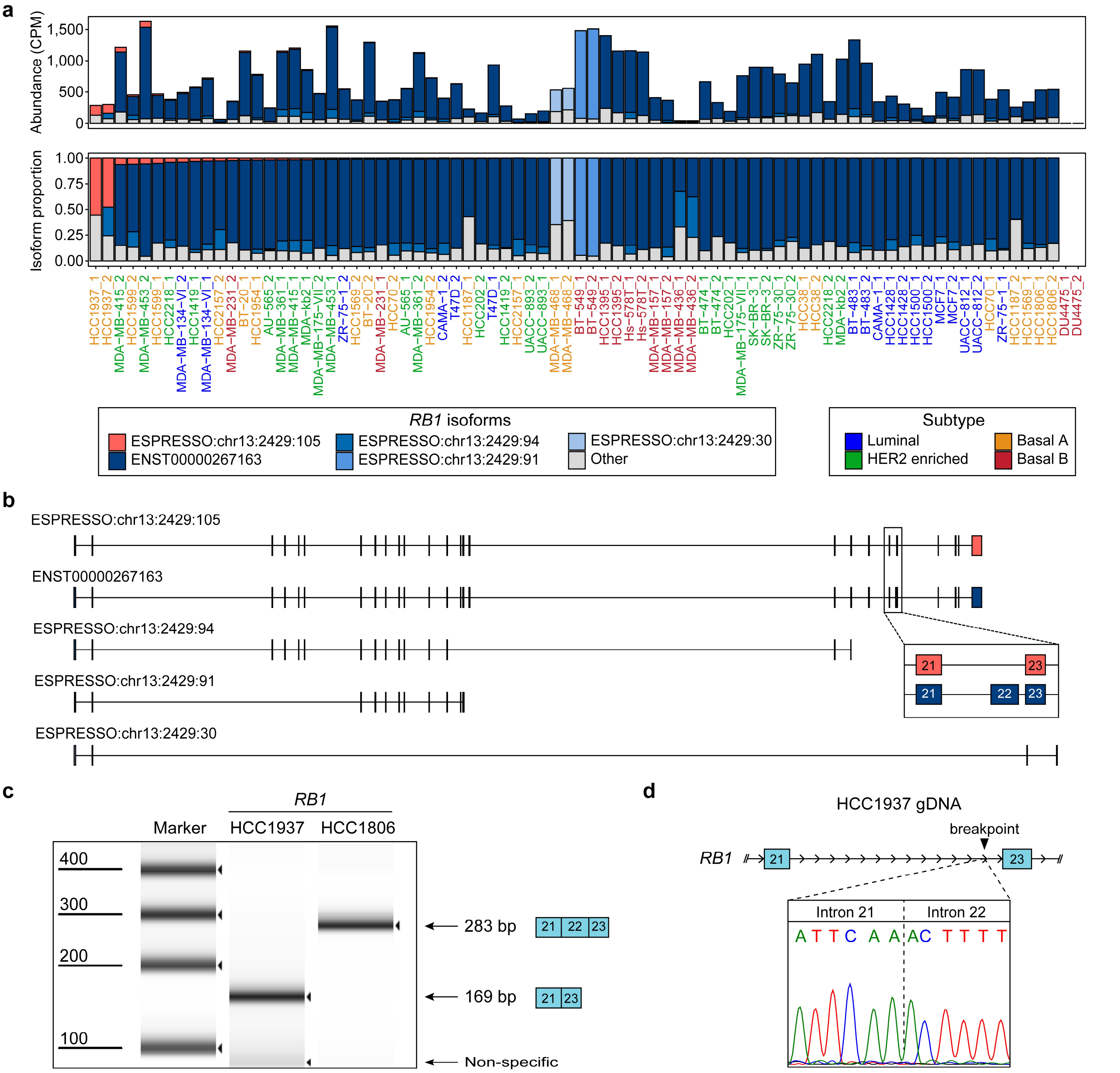

Supplementary Figure 8. A novel aberrant *RB1* transcript isoform resulting from a genomic deletion containing exon 22 is the predominant isoform in the HCC1937 cell line. a, Stacked barplots showing estimated abundances (upper panel) and proportions (lower panel) of *RB1* transcript isoforms discovered by TEQUILA-seq in 40 cell lines. Red bar: isoform of interest (ESPRESSO:chr13:2429:105); navy bar: canonical isoform (ENST00000267163); lighter blue bars: 3 other most abundant *RB1* isoforms; gray bars: remaining *RB1* isoforms. **b,** Structures of *RB1* transcript isoforms. Boxes: exons. Line segments: introns. **c,** RT-PCR validation of splice variants containing exons 21 and 23 of *RB1* in HCC1937 and HCC1806 (control) cell lines. The 283-bp product corresponds to the canonical transcript isoform of *RB1* in HCC1806 (control), and the 169-bp product corresponds to the predominant novel transcript isoform resulting from exon 22 skipping in HCC1937. Each RT-PCR was repeated twice and yielded the same PCR products. **d,** Sanger sequencing identifies a 178-bp genomic deletion in HCC1937 containing *RB1* exon 22. Sequencing results for the *RB1* gDNA amplicons from HCC1937 are shown. Breakpoints of the deletion are located in introns 21 and 22 of *RB1*. Dashed line in partial sequence chromatogram: breakpoint.

Supplementary Figure 9

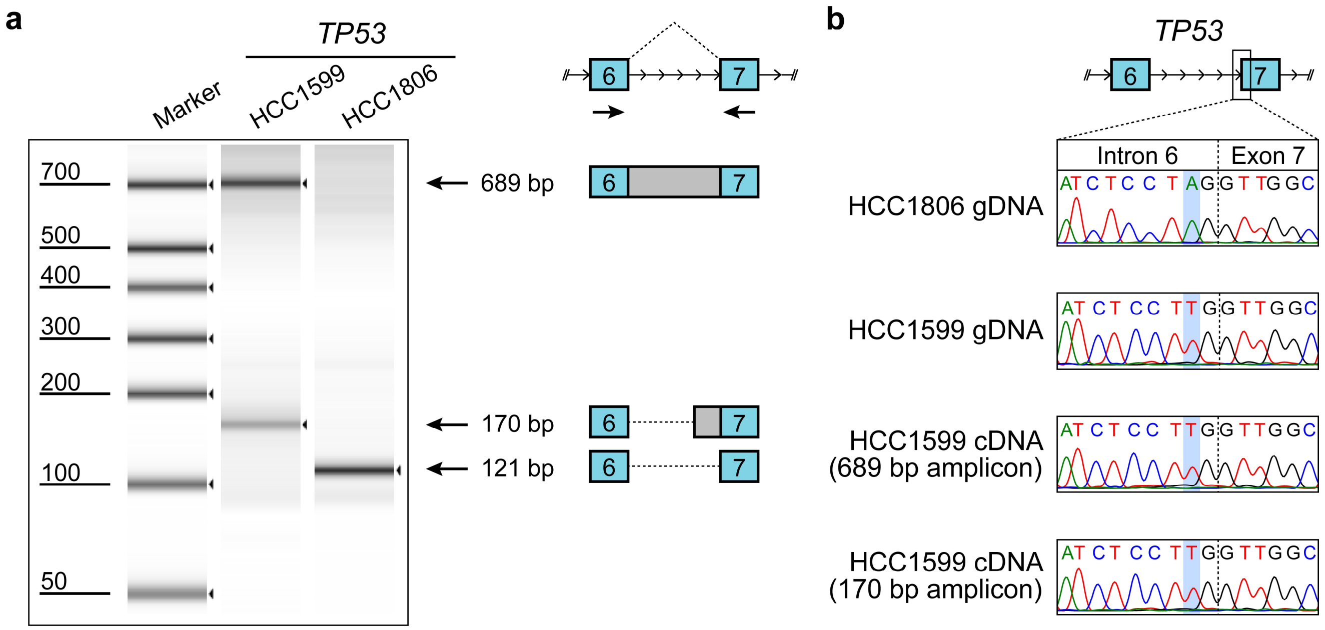

Supplementary Figure 9. Confirmation of a splice site-disrupting mutation causing *TP53* splice variants in the HCC1599 cell line. a, RT-PCR validation of splice variants containing exons 6 and 7 of *TP53* in HCC1599 and HCC1806 (control) cell lines. The 121-bp product corresponds to the canonical transcript isoform of *TP53* in HCC1806 (control), the 689-bp product corresponds to the predominant novel transcript isoform containing a retained intron in HCC1599, and the 170-bp product corresponds to a second, minor novel transcript isoform using a novel 3' splice site in HCC1599. Each RT-PCR was repeated twice and yielded the same PCR products. **b,** Sanger sequencing identifies a 3'-splice site mutation (A>T) of *TP53* intron 6 in HCC1599. Sequencing results are shown for the *TP53* gDNA amplicons from the HCC1599 and HCC1806 (control) cell lines, as well as the *TP53* cDNA amplicons from the HCC1599 cell line. HCC1806 harbors the wild type 3'-splice site dinucleotide AG, whereas HCC1599 harbors a mutated 3'-splice site dinucleotide TG. Dashed line in partial sequence chromatogram: boundary between intron 6 and exon 7.

Supplementary Figure 10

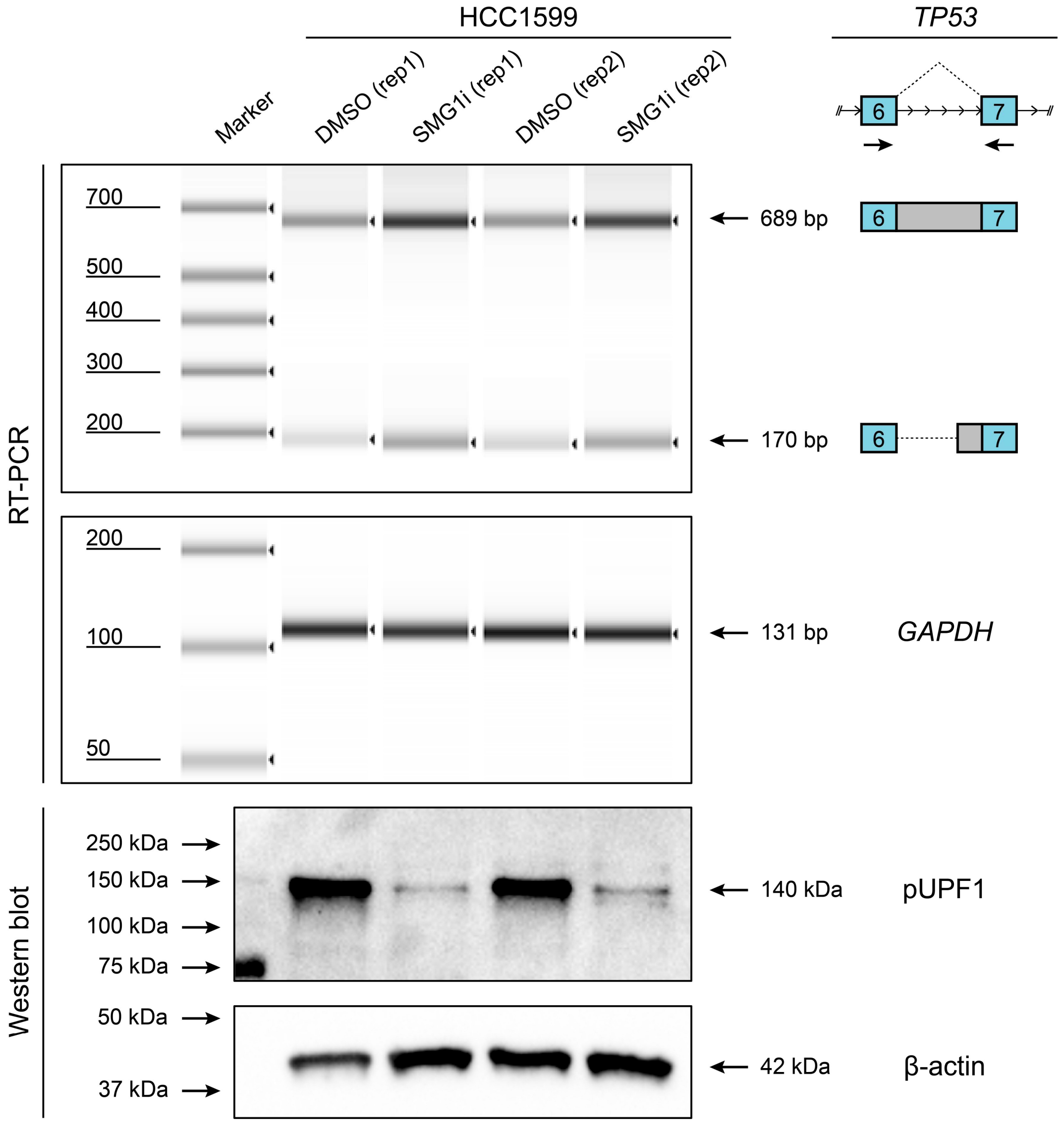

Supplementary Figure 10. Elevated transcript expression levels of two PTC-containing transcript isoforms of *TP53* upon NMD inhibition in HCC1599 cells, as indicated by RT-PCR analysis. The 689-bp product corresponds to the predominant novel transcript isoform containing a retained intron, while the 170-bp product corresponds to a second, minor novel transcript isoform using a novel 3' splice site. *GAPDH* was used as the loading control for RT-PCR. Western blot analysis of phosphorylated UPF1 (pUPF1) was used to measure NMD activity, and β-actin was used as the loading control for Western blot. Each RT-PCR or Western blot was repeated twice and yielded consistent results.

Supplementary Figure 11

a

Probe yield (µg)

Template oligo pool	1 hr	2 hr	4 hr	8 hr	16 hr
	**Incubation time**				
2 ng	**2.96**	**7.58**	**16.86**	**21.86**	**25.95**
4 ng	**4.02**	**9.06**	**20.16**	**24.33**	**26.87**
6 ng	**4.89**	**10.46**	**22.88**	**26.67**	**28.26**
8 ng	**5.60**	**11.43**	**24.84**	**27.74**	**28.11**
10 ng	**6.35**	**12.14**	**26.60**	**28.01**	**28.28**

b

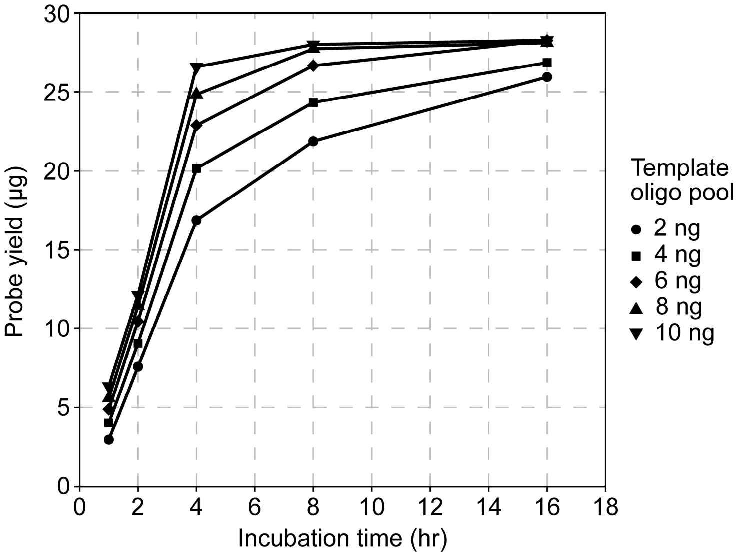

Supplementary Figure 11. Probe yield of individual synthesis reactions measured by Nanodrop. a and **b** show probe yield from different amounts of template oligo pool (ranging from 2 ng to 10 ng) and incubation times (ranging from 1 hr to 16 hr).

Supplementary Figure 12

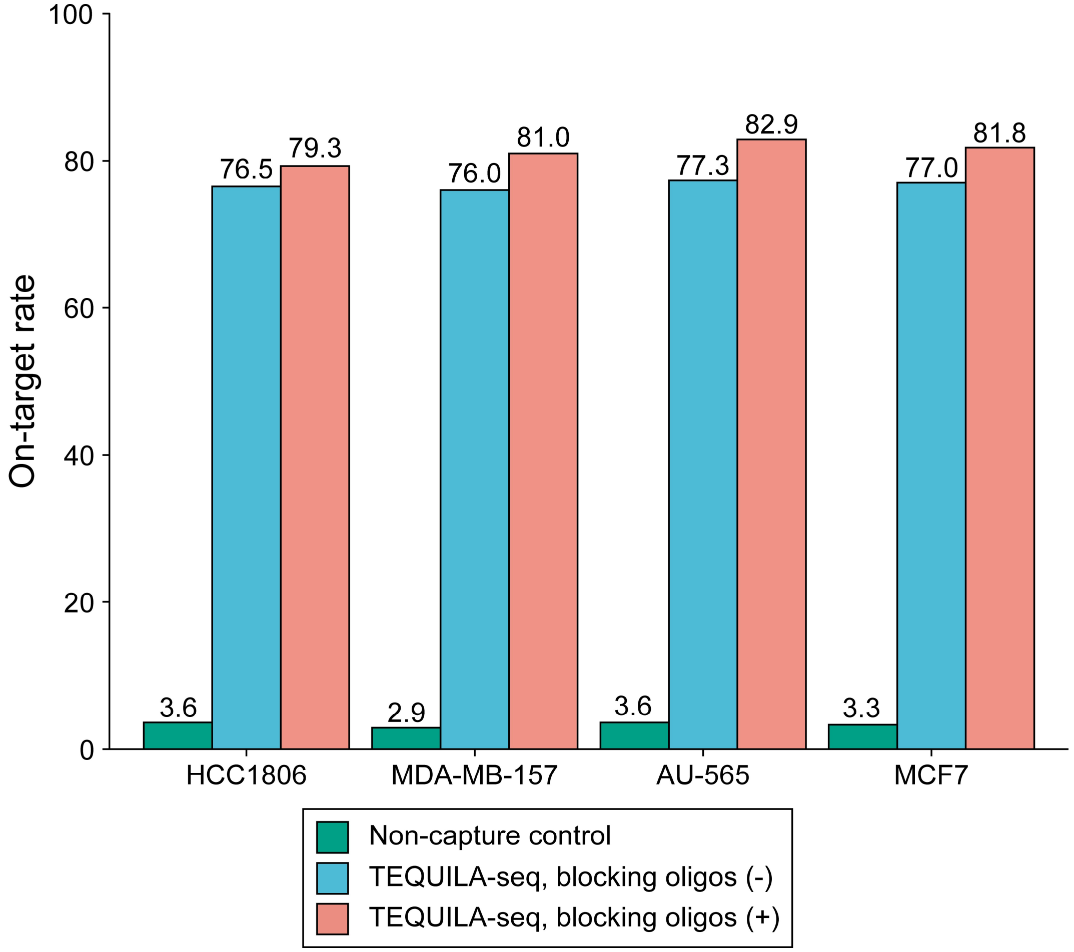

Supplementary Figure 12. Target enrichment of 468 actionable cancer genes in 4 breast cancer cell lines using TEQUILA-seq with or without blocking oligos. For each cell line, TEQUILA-seq (with or without blocking oligos) and whole-transcriptome nanopore 1D cDNA sequencing (non-capture control) libraries were prepared from the same biological replicate. Each bar shows the percentage of reads mapped to the 468 genes.

Supplementary Figure 13

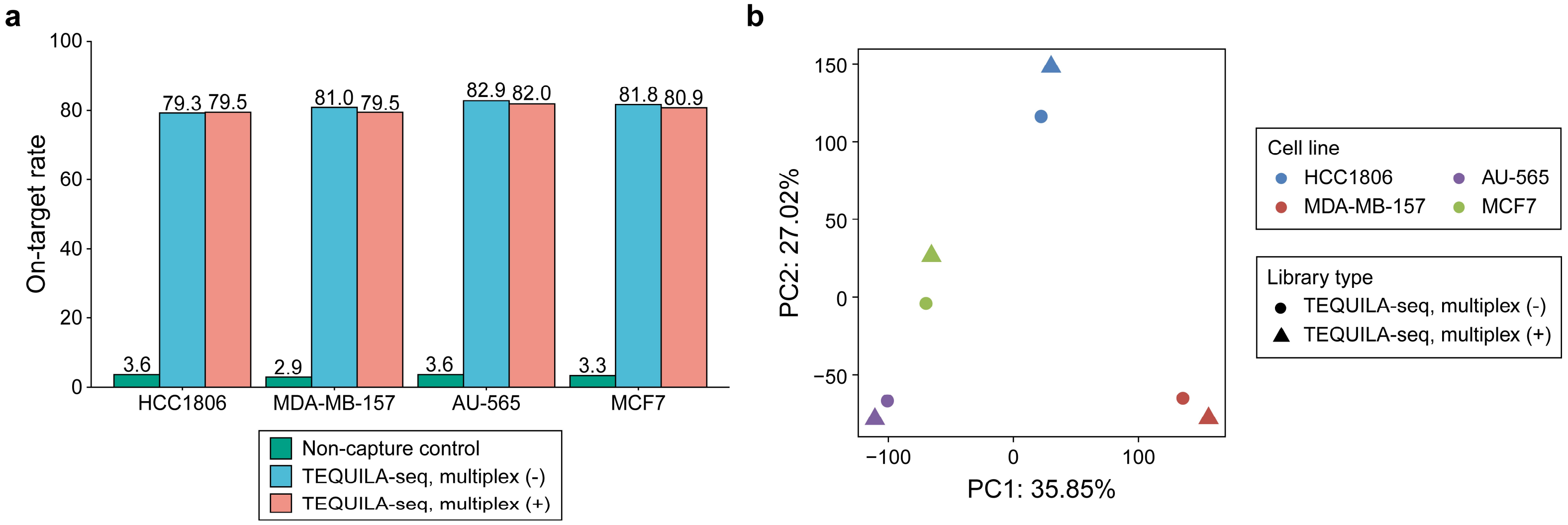

Supplementary Figure 13. Barcoding and multiplexing TEQUILA-seq libraries using the nanopore native barcoding kit. a, Percentage of reads mapped to 468 actionable cancer genes in 4 breast cancer cell lines using TEQUILA-seq (with or without multiplexing) and whole-transcriptome nanopore 1D cDNA sequencing (non-capture control). **b,** Principal component analysis using estimated abundances of all transcript isoforms across 468 genes in 4 cell lines. Each data point represents one TEQUILA-seq sample for a cell line.
