# Supplementary material for: TEQUILA-seq: a versatile and low-cost method for targeted long-read RNA sequencing
Source: Nat Commun. 2023 Aug 8;14:4760. doi: 10.1038/s41467-023-40083-6 (PMC10409798; doi:10.1038/s41467-023-40083-6)
Supplement: Supplementary file 3 — Description of Additional Supplementary Files [file 41467_2023_40083_MOESM3_ESM.pdf]

Description of Additional Supplementary Files

File Name: Supplementary Data 1
Description: Reagent cost for synthesizing TEQUILA probes.

File Name: Supplementary Data 2
Description: Cost comparison between IDT xGen Lockdown probes and TEQUILA probes.

File Name: Supplementary Data 3
Description: Test panel of 10 human brain genes.

File Name: Supplementary Data 4
Description: Summary statistics for targeted long-read sequencing of human brain total RNA.

File Name: Supplementary Data 5
Description: SIRV-Set 4 modules used for targeted long-read sequencing.

File Name: Supplementary Data 6
Description: Summary statistics for sequencing protocol comparison.

File Name: Supplementary Data 7
Description: Panel of 221 genes encoding human splicing factors (SFs).

File Name: Supplementary Data 8
Description: Summary statistics for short-read RNA-seq of SH-SY5Y cell line.

File Name: Supplementary Data 9
Description: Panel of 468 actionable cancer genes.

File Name: Supplementary Data 10
Description: Panel of 40 breast cancer cell lines.

File Name: Supplementary Data 11
Description: Sequencing summary statistics for 40 breast cancer cell lines.

File Name: Supplementary Data 12
Description: Summary of identified subtype-associated transcript isoforms of 468 actionable cancer genes in 40 breast cancer cell lines.

File Name: Supplementary Data 13
Description: Cell culture conditions recommended by ATCC for 40 breast cancer cell lines.

File Name: Supplementary Data 14
Description: List of oligonucleotides, primers, and probe sequences.

File Name: Supplementary Data 15
Description: Summary of nanopore RNA-seq libraries.
