# Supplementary material for: TEQUILA-seq: a versatile and low-cost method for targeted long-read RNA sequencing
Source: Nat Commun. 2023 Aug 8;14:4760. doi: 10.1038/s41467-023-40083-6 (PMC10409798; doi:10.1038/s41467-023-40083-6)
Supplement: Supplementary file 5 — Reporting Summary [file 41467_2023_40083_MOESM5_ESM.pdf]

nature portfolio

Corresponding author(s): Yi Xing, Lan Lin

Last updated by author(s): Jun 8, 2023

Reporting Summary

Nature Portfolio wishes to improve the reproducibility of the work that we publish. This form provides structure for consistency and transparency in reporting. For further information on Nature Portfolio policies, see our Editorial Policies and the Editorial Policy Checklist.

Statistics

For all statistical analyses, confirm that the following items are present in the figure legend, table legend, main text, or Methods section.

n/a	Confirmed	
☐	☒	The exact sample size (*n*) for each experimental group/condition, given as a discrete number and unit of measurement
☐	☒	A statement on whether measurements were taken from distinct samples or whether the same sample was measured repeatedly
☐	☒	The statistical test(s) used AND whether they are one- or two-sided<br>*Only common tests should be described solely by name; describe more complex techniques in the Methods section.*
☒	☐	A description of all covariates tested
☐	☒	A description of any assumptions or corrections, such as tests of normality and adjustment for multiple comparisons
☐	☒	A full description of the statistical parameters including central tendency (e.g. means) or other basic estimates (e.g. regression coefficient) AND variation (e.g. standard deviation) or associated estimates of uncertainty (e.g. confidence intervals)
☐	☒	For null hypothesis testing, the test statistic (e.g. *F*, *t*, *r*) with confidence intervals, effect sizes, degrees of freedom and *P* value noted<br>*Give P values as exact values whenever suitable.*
☒	☐	For Bayesian analysis, information on the choice of priors and Markov chain Monte Carlo settings
☒	☐	For hierarchical and complex designs, identification of the appropriate level for tests and full reporting of outcomes
☐	☒	Estimates of effect sizes (e.g. Cohen's *d*, Pearson's *r*), indicating how they were calculated

Our web collection on statistics for biologists contains articles on many of the points above.

Software and code

Policy information about availability of computer code

Data collection: Guppy (version 4.0.15) was used to perform basecalling of raw nanopore sequencing data (fast5 format). In the technical refinement section, basecalling was performed in super-accurate mode using Guppy (v6.4.2) with the 'dna_r9.4.1_450bps_sup.cfg' config file for R9.4.1 and the 'dna_r10.4.1_e8.2_260bps_sup.cfg' config file for R10.4.1 chemistry. Basecalled reads from multiplexed nanopore sequencing data were also demultiplexed using Guppy (v6.4.2) with the parameter: '--barcode_kits SQK-NBD114-24'.

Data analysis: Basecalled reads were mapped to the GRCh37/hg19 reference genome using minimap2 (version 2.17). Full-length transcript isoforms were discovered and quantified from long-read alignment files using ESPRESSO (version 1.2.2). Illumina TruSeq Stranded mRNA short-read RNA-seq data was aligned to the GRCh37/hg19 reference genome using STAR (version 2.6.1d). Exon skipping events were discovered and quantified from short-read alignment files using rMATS (version 4.1.1). The scripts used for processing, analyzing, and visualizing TEQUILA-seq data are publicly available on GitHub (https://github.com/Xinglab/TEQUILA-seq). RT-PCR results were analyzed using Tapestation Analysis Software 4.1.1. Blots were imaged and analyzed on a ChemiDoc XRS+ system with Image Lab software (Bio-Rad, #1708265).

For manuscripts utilizing custom algorithms or software that are central to the research but not yet described in published literature, software must be made available to editors and reviewers. We strongly encourage code deposition in a community repository (e.g. GitHub). See the Nature Portfolio guidelines for submitting code & software for further information.

Data

Policy information about availability of data

All manuscripts must include a data availability statement. This statement should provide the following information, where applicable:

- Accession codes, unique identifiers, or web links for publicly available datasets
- A description of any restrictions on data availability
- For clinical datasets or third party data, please ensure that the statement adheres to our policy

Raw and processed data from Illumina short-read RNA-seq and nanopore long-read RNA-seq were uploaded to GEO under accession number GSE213984 (https://www.ncbi.nlm.nih.gov/geo/query/acc.cgi?acc=GSE213984). Transcript annotations from GENCODE v34 were downloaded from https://www.gencodegenes.org/human/release_34lift37.html. Canonical transcript isoform annotations were obtained from the Ensembl database (Release 100, April 2020).

Human research participants

Policy information about studies involving human research participants and Sex and Gender in Research.

Reporting on sex and gender	*Not applicable*
Population characteristics	*Not applicable*
Recruitment	*Not applicable*
Ethics oversight	*Not applicable*

Note that full information on the approval of the study protocol must also be provided in the manuscript.

Field-specific reporting

Please select the one below that is the best fit for your research. If you are not sure, read the appropriate sections before making your selection.

☒ Life sciences ☐ Behavioural & social sciences ☐ Ecological, evolutionary & environmental sciences

For a reference copy of the document with all sections, see nature.com/documents/nr-reporting-summary-flat.pdf

Life sciences study design

All studies must disclose on these points even when the disclosure is negative.

Sample size	One human brain total RNA sample (Clontech, Cat. #636530, Lot. #2006022), one SH-SY5Y human neuroblastoma cell line (ATCC, #CRL-2266), and 40 breast cancer cell lines in the ATCC Breast Cancer Cell Panel (ATCC, 30-4500K). The sample sizes were chosen based on common practices and resource availability. Specifically, we analyzed all 40 breast cancer cell lines in the ATCC Breast Cancer Cell Panel (ATCC, 30-4500K).
Data exclusions	No data were excluded from this study.
Replication	We prepared TEQUILA-seq, xGen Lockdown-seq, and nanopore 1D cDNA-seq libraries (each with 3 technical replicates) from the same human brain total RNA sample (Clontech, Cat. #636530, Lot. #2006022); Illumina TruSeq Stranded mRNA, nanopore direct RNA-seq, nanopore 1D cDNA-seq, and TEQUILA-seq libraries from 3 biological replicates of the SH-SY5Y human neuroblastoma cell line (ATCC, #CRL-2266); TEQUILA-seq libraries from 40 breast cancer cell lines (ATCC, 30-4500K), each with 2 biological replicates. Biological replicates were constructed from RNA samples derived from different cell cultures and different RNA extractions. Technical replicates were constructed separately from RNA samples derived from the same cell cultures and RNA extractions. All attempts at replication were successful. All experiments other than those mentioned here in the reporting summary were replicated twice and performed independently.
Randomization	Randomization was not relevant to this study because our study does not involve the assignment of test subjects or treatments.
Blinding	Blinding was not relevant to this study because our study does not involve the assignment of test subjects or treatments.

Reporting for specific materials, systems and methods

We require information from authors about some types of materials, experimental systems and methods used in many studies. Here, indicate whether each material, system or method listed is relevant to your study. If you are not sure if a list item applies to your research, read the appropriate section before selecting a response.

Materials & experimental systems

n/a	Involved in the study
☐	☒ Antibodies
☐	☒ Eukaryotic cell lines
☒	☐ Palaeontology and archaeology
☒	☐ Animals and other organisms
☒	☐ Clinical data
☒	☐ Dual use research of concern

Methods

n/a	Involved in the study
☒	☐ ChIP-seq
☒	☐ Flow cytometry
☒	☐ MRI-based neuroimaging

Antibodies

Antibodies used

Phospho-Upf1 (Ser1127) antibody (Sigma-Aldrich, #07-1016), β-actin loading control monoclonal antibody (BA3R) (Invitrogen, #MA5-15739), HRP-conjugated goat anti-rabbit IgG (Agilent Technologies, #P044801-2), and goat anti-mouse IgG secondary antibody (Invitrogen, #PI32430).

Validation

Phospho-Upf1 (Ser1127) antibody (Sigma-Aldrich, #07-1016): immunoblot signal at expected size was observed for endogenous Upf1 in HCC1599 whole cell lysates. Usage was also published in Zhao et al. The RNA quality control pathway nonsense-mediated mRNA decay targets cellular and viral RNAs to restrict KSHV. Nat Commun 11, 3345 (2020). https://doi.org/10.1038/s41467-020-17151-2.

β-actin loading control monoclonal antibody (BA3R) (Invitrogen, #MA5-15739): immunoblot signal at expected size was observed for endogenous β-actin in HCC1599 whole cell lysates. Usage was also published in over 230 papers to date (6/8/2023), see references from the supplier's website (https://www.thermofisher.com/antibody/product/beta-Actin-Loading-Control-Antibody-clone-BA3R-Monoclonal/MA5-15739).

Eukaryotic cell lines

Policy information about cell lines and Sex and Gender in Research

Cell line source(s)

SH-SY5Y human neuroblastoma cells (ATCC, #CRL-2266); Breast Cancer Cell Panel (ATCC, 30-4500K™) including BT-20, HCC38, HCC70, HCC1187, HCC1569, HCC1599, HCC1806, HCC1937, HCC1954, HCC2157, MDA-MB-468, BT-549, HCC1395, Hs-578T, MDA-MB-157, MDA-MB-231, MDA-MB-436, DU4475, AU-565, BT-474, HCC202, HCC1419, MDA-MB-175-VII, MDA-MB-361, MDA-MB-415, MDA-MB-453, SK-BR-3, UACC-893, ZR-75-30, HCC2218, MDA-kb2, BT-483, CAMA-1, HCC1428, HCC1500, MCF7, MDA-MB-134-VI, T47D, UACC-812, and ZR-75-1.

Authentication

SH-SY5Y cell line was authenticated using short tandem repeat (STR) analysis. Breast cancer cell lines were authenticated using STR analysis by the supplier.

Mycoplasma contamination

Cells were verified to be mycoplasma-free by the Lonza MycoAlert assay.

Commonly misidentified lines
(See ICLAC register)

No commonly misidentified cell lines were used in this study.
